# Maturing Human CD127+ CCR7+ PDL1+ Dendritic Cells Express AIRE in the Absence of Tissue Restricted Antigens

**DOI:** 10.3389/fimmu.2018.02902

**Published:** 2019-01-14

**Authors:** Joannah R. Fergusson, Michael D. Morgan, Melanie Bruchard, Leonie Huitema, Balthasar A. Heesters, Vincent van Unen, Jan Piet van Hamburg, Nicole N. van der Wel, Daisy Picavet, Frits Koning, Sander W. Tas, Mark S. Anderson, John C. Marioni, Georg A. Holländer, Hergen Spits

**Affiliations:** ^1^Department of Experimental Immunology, Academic Medical Center, Amsterdam, Netherlands; ^2^Wellcome Sanger Institute, Hinxton, United Kingdom; ^3^Department of Rheumatology & Clinical Immunology and Department of Experimental Immunology, Amsterdam Infection & Immunity Institute, University of Amsterdam, Amsterdam, Netherlands; ^4^Amsterdam Rheumatology & immunology Center (ARC), Academic Medical Center, Amsterdam, Netherlands; ^5^Leiden University Medical Center, Leiden, Netherlands; ^6^EMCA, Medical Biology, Academic Medical Center, Amsterdam, Netherlands; ^7^UCSF Diabetes Center, University of California, San Francisco, San Francisco, CA, United States; ^8^European Molecular Biology Laboratory - European Bioinformatics Institute (EMBL-EBI), Hinxton, United Kingdom; ^9^Cancer Research UK Cambridge Institute, University of Cambridge, Cambridge, United Kingdom; ^10^Laboratory of Developmental Immunology, Weatherall Institute of Molecular Medicine and Department of Paediatrics, University of Oxford, Oxford, United Kingdom

**Keywords:** dendritic cells, AIRE, PDL1, maturation, tissue restricted antigen

## Abstract

Expression of the Autoimmune regulator (AIRE) outside of the thymus has long been suggested in both humans and mice, but the cellular source in humans has remained undefined. Here we identify AIRE expression in human tonsils and extensively analyzed these “extra-thymic AIRE expressing cells” (eTACs) using combinations of flow cytometry, CyTOF and single cell RNA-sequencing. We identified AIRE+ cells as dendritic cells (DCs) with a mature and migratory phenotype including high levels of antigen presenting molecules and costimulatory molecules, and specific expression of CD127, CCR7, and PDL1. These cells also possessed the ability to stimulate and re-stimulate T cells and displayed reduced responses to toll-like receptor (TLR) agonists compared to conventional DCs. While expression of *AIRE* was enriched within CCR7+CD127+ DCs, single-cell RNA sequencing revealed expression of *AIRE* to be transient, rather than stable, and associated with the differentiation to a mature phenotype. The role of AIRE in central tolerance induction within the thymus is well-established, however our study shows that *AIRE* expression within the periphery is not associated with an enriched expression of tissue-restricted antigens (TRAs). This unexpected finding, suggestive of wider functions of AIRE, may provide an explanation for the non-autoimmune symptoms of APECED patients who lack functional AIRE.

## Introduction

Autoimmune regulator (AIRE) has primarily received attention due to its expression within the thymus and role in tolerance induction. Two parallel observations led to the identification of AIRE as a master regulator of central tolerance; firstly the presence of transcripts for peripheral antigens within the thymus (1) and secondly the identification of a genetic locus with transcriptional activity responsible for the multi-organ autoimmune disease, known as autoimmune polyendocrinopathy candidiasis ectodermal dystrophy (APECED) (2–5). A seminal study by Anderson et al. (6) provided the link between these observations. Using AIRE-deficient mice this study demonstrated the role of AIRE in promoting tissue-specific gene expression within the thymus and a related reduction in the self-reactivity of peripheral T cells. Expression of AIRE and associated tissue restricted antigens (TRAs) are largely restricted to a specific population of epithelial cells within the medulla (mTEC) (1), where developing thymocytes are screened for self-reactivity and deleted before release into the periphery. Together, these findings established thymic AIRE as the master transcription factor for central tolerance induction during T cell development.

Several reports have also detected expression of AIRE outside the thymus (3, 6). However, reports have been conflicting and the cellular source controversial, with reports in both stromal (7, 8) and hematopoetic (9–11) populations. Recently, a population of AIRE expressing cells within the secondary lymphoid organs of mice were identified (12). These cells were bone marrow-derived yet with low surface CD45 levels, potentially reconciling reports of AIRE expression in both stromal and hematopoetic lineages. These AIRE-expressing cells formed a distinct Major Histocompatibility Class II (MHCII)^hi^ antigen presenting population with the ability to functionally inactivate CD4+ T cells (12). However, the existence of a corresponding extra-thymic AIRE expressing cell (eTAC) in humans has yet to be firmly established, or the identity of such cells described. Here, we demonstrate AIRE to be expressed outside the human thymus, and extensively characterize these cells within human lymphoid organs. We find these cells to be CD127+ DCs with a mature CCR7+PDL1+ phenotype that express AIRE during their maturation.

## Results

### Extra-Thymic Expression of AIRE in Human Tonsil

**Figure 1:**
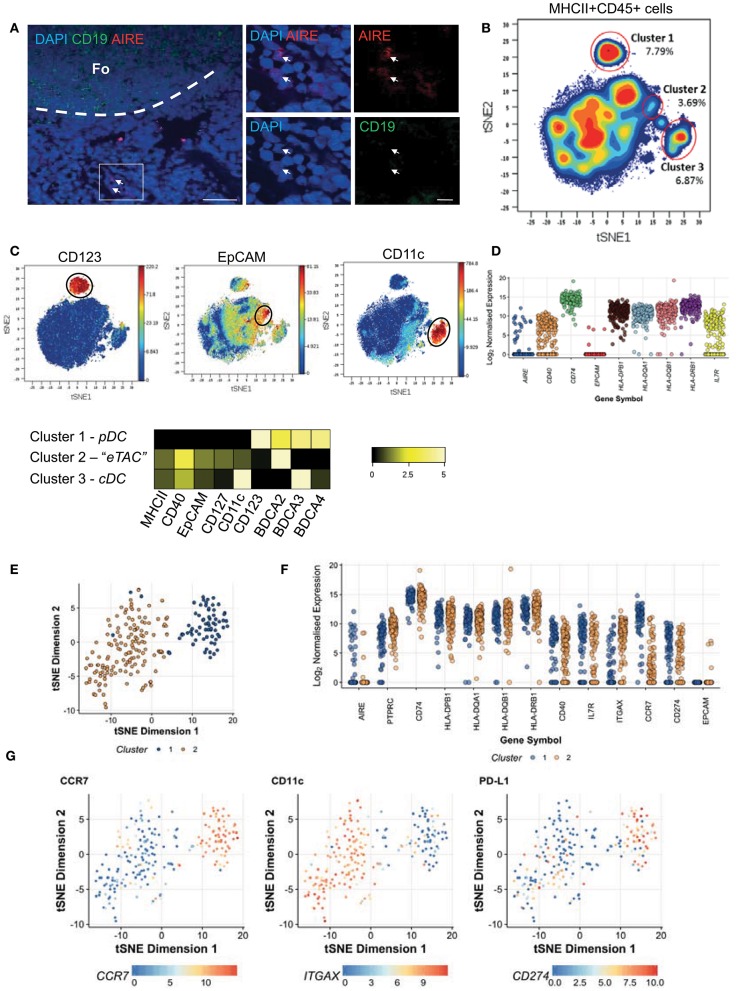
Extrathymic AIRE expressing cells are present in human tonsil. **(A)** Human tonsil sections stained by immunofluorescence for DAPI (blue), AIRE (red), and CD19 (green). The dashed line indicates the border of the lymphoid follicle (Fo) and scale bars indicating 200 μM (left) or 20 μM (right) are shown; representative of 2 donors tested. **(B)** t-SNE analysis of all HLA-DR+ CD45+ cells from pooled human tonsils (*n* = 2) generated by considering all 33 markers measured, shown as a contour plot **(C)** t-SNE analysis as in **(B)** colored according to the intensity of expression of CD123, EpCAM, and CD11c. Heatmap indicates the log_2_ ratio of means for each marker normalized to the minimum cluster expression (row minimum) according to the specific cell clusters identified and indicated in **(B) (D)** CD45+MHCII+CD40+CD127+EpCAM+ cells were sequenced as single cells and log_2_ normalized transcript levels of sorting markers and AIRE are shown **(E)** t-SNE analysis of highly variable genes as measured by RNA-Seq across both donors revealing 2 distinct clusters; cluster 1 (blue dots) and cluster 2 (orange) **(F)** Log_2_ normalized transcript expression levels for AIRE and for defining markers CD45 (PTPRC), CD74, MHCII (HLA-DPB1, HLA-DQA1, HLA-DQB1, HLA-DRB1), CD40, CD127 (IL7R), CD11c (ITGAX) as well as CCR7 and PDL1 according to the clusters identified **(G)** Expression levels of CCR7, PDL1 (CD274), and CD11c (ITGAX) transcripts per cell overlaid on the t-SNE plot. See also Supplementary Figure 1.

By confocal imaging AIRE expressing cells could be identified within human peripheral tonsil tissue (Figure 1A) in addition to those within the human thymus, which has been well-defined (Supplementary Figure 1A). These extra-thymic AIRE positive cells of the tonsil were localized within the T cell zone, at the boundary between the T cell paracortex and B cell follicles. eTACs have previously been described in mice as a bone marrow-derived CD45+ and MHCII+ APC population which expresses the epithelial marker EpCAM (12). To identify the potential human equivalents of eTACs in mice we performed a screen of 33 surface markers using Cytometry by Time-of-Flight (CyTOF). Given the low levels of AIRE expression in the periphery (6, 7), and the failure of in-house conjugated AIRE antibodies to detect AIRE (data not shown), the panel was designed to include those surface markers which define mouse eTACs, and other innate and antigen-presenting cell types. With t-distributed stochastic neighbor embedding (t-SNE) of all tonsil CD45+MHCII+ cells, and by using color as a third dimension to visualize the intensity of expression of specific markers, distinct clusters relating to conventional dendritic cells (CD11c; cDC) and plasmacytoid dendritic cells (CD123; pDC) were clearly visible (Figures 1B,C). Mouse eTACs have previously been characterized by high expression of EpCAM amongst MHCII+ leukocytes; (12) therefore, we used EpCAM expression to identify a third cluster on the t-SNE plot putatively representing human eTACs. These cells shared expression of markers with both cDC and pDC, but uniquely displayed expression of CD127 (Figure 1C).

Therefore, we initially defined human eTACs as CD45+MHCII+CD40+CD127+EpCAM+. To determine whether this cluster represented the human equivalent of mouse eTACs, and to characterize this population further, we sorted these cells from 2 human tonsils (Supplementary Figure 1B) for expression profiling by single-cell RNA-sequencing. Comparison of the distribution in sequencing depth per cell for each donor showed no systemic differences between the 2 donors (Supplementary Figure 1C).

We detected abundant mRNA for *IL7RA* (CD127), HLA class II loci and the associated *CD74*, and *CD40*. Intriguingly, transcripts for EpCAM were detected in only 4/217 (1.8%) cells. *AIRE* mRNA was variably expressed, and restricted to only a small proportion of cells (25/217 cells; 11.5%) (Figure 1D); indicating the potential for a more refined phenotype to capture these cells. To identify cells with the greatest *AIRE* expression the high dimensional transcriptomes of donor cells were embedded into 2 dimensions using t-SNE, which revealed the appearance of 2 clusters. Cells were computationally assigned to distinct groups using hierarchical clustering (Figure 1E). These clusters did not correspond to the individual donors, as the 2 donors were uniformly represented across both clusters (Supplementary Figure 1D). *AIRE* expression was primarily restricted to the smaller group of cells (Figure 1F –blue cells). To focus on reliable surface markers for these AIRE-expressing cells, we then performed differential expression testing between these 2 clusters (Supplementary Figures 1E,F). Of the genes that were robustly differentially expressed between the *AIRE*-enriched cluster and the remaining cells, *CCR7* was most specifically and strongly up-regulated (Figures 1F,G and Supplementary Figure 1F). In addition we noted that the inhibitory receptor PDL1 was also enriched in these cells, while CD11c was relatively down-regulated, as illustrated in Figure 1G.

### AIRE Is Expressed by a CD45+ DC Population Expressing CCR7

**Figure 2:**
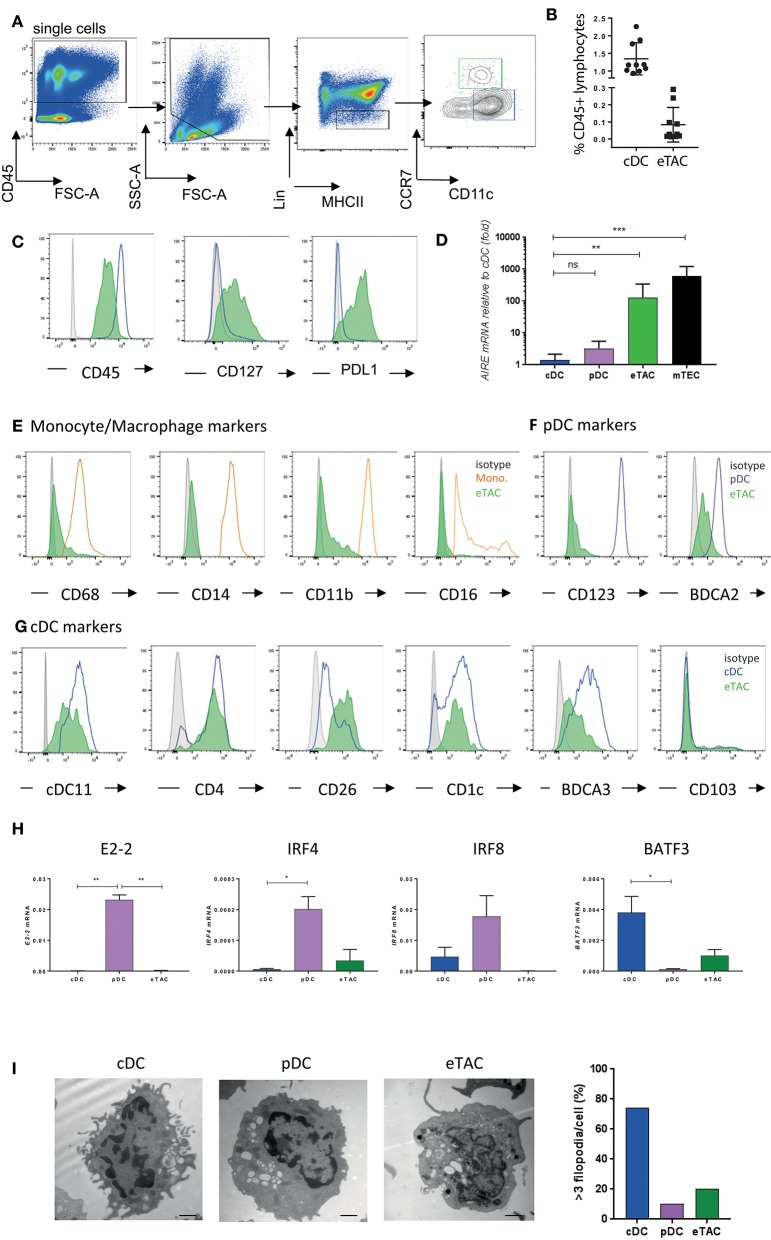
AIRE is expressed by a CD45+MHCII+ dendritic cell population expressing CCR7 and CD127 **(A)** Gating strategy based upon CD45, MHCII, Lin (CD3, CD19, TCRαβ, TCRγδ, CD123, CD14, CD16, CD94, CD34), and CCR7. Gating for eTAC (green) and CCR7-CD11c+ conventional DC (blue) are indicated **(B)** Frequency of eTACs and cDCs as percentage of CD45+ lymphocytes from tonsil as gated in A (*n* = 10) **(C)** Histograms display levels of CD45, CD127, and PDL1 in cDCs (blue line) and eTACs (filled green) compared to isotype (gray), representative of at least 10 donors **(D)** *AIRE* mRNA expression in tonsillar pDCs (*n* = 5) and eTACs (*n* = 9), and postnatal thymic mTECs (*n* = 4) relative to tonsillar cDCs (*n* = 9), normalized to β-actin levels, (mean ± SD ns = not significant, **p < 0.005, ***p < 0.005 by Kruskal-Wallis with Dunn's multiple comparison to cDCs) **(E–G)** Flow cytometric analysis of expression of various monocyte [**(E)**; orange] **(E)**, pDC [(**F)**; purple] and cDC [**(G)**; blue] markers by tonsil eTACs (filled green) compared to isotype (gray) **(H)** mRNA expression for E2-2, IRF4, IRF8, and BATF3 in eTACs, pDCs, and cDCs (*n* = 3), normalized to β-actin levels (mean ± SD *p < 0.05, **p < 0.005, ***p < 0.005 by ANOVA with Tukey's multiple comparisons test) **(I)** Representative images of cDCs, pDCs, and eTACs from human tonsil imaged by electron microscopy. Scale bars of 1 μM are shown. Bar graph indicates percentage of cells in each subset with >3 filopodia. See also Supplementary Figures 2, 3.

We subsequently used these clustering and gene expression findings to develop a FACS-based gating strategy for the identification of AIRE-expressing cells (Figure 2A). With the exclusion of other cell types using an extensive lineage cocktail, CCR7+ cells that expressed intermediate levels of CD11c could be identified amongst MHCII+ antigen-presenting cells. This population was distinct from cDCs but found at a much lower frequency (eTACs 0.08 ± 0.1% vs. cDCs 1.344 ± 0.4%). These cells remained uniquely CD127 positive and expressed cDCs (Figure 2B), although at lower levels than cDCs, (Figure 2B), in addition to expressing PDL1 (Figure 2C). This was therefore concordant with results from our single-cell RNA-sequencing experiment. Transcripts for *AIRE* within this sorted population were highly enriched, with greater enrichment than seen in the original population sorted on EpCAM (Supplementary Figure 2A). Expression was variable between donors; nevertheless we observed ~100-fold greater expression compared to cDC. However, these levels still represent an order of magnitude lower than those observed in mTECs (Figure 2D, gating strategy shown in Supplementary Figure 2B). Henceforward we use the term eTAC to denote this extra-thymic AIRE-enriched cell population of CCR7+CD127+MHCII+ cells.

By the same gating strategy, a population of Lin–CD127+MHCII+CCR7+ cells, which were enriched for *AIRE* mRNA, were identified within the postnatal thymus (Supplementary Figure 2C). These CD45+ cells were distinct from mTEC, and displayed a similar phenotype to AIRE-enriched cells within the tonsil, including expression of PDL1 (Supplementary Figures 2D–G).

Detailed phenotypic analysis of this CCR7+ AIRE-enriched population within tonsils demonstrated the absence of classical monocyte markers CD68, CD16, CD14, and CD11b at the protein level (Figure 2E). These cells were likewise negative for CD123, a marker of pDCs, but did express low levels of BDCA2 protein (Figure 2F). The greatest similarity, based on protein marker expression, was with cDCs. AIRE-enriched cells expressed several markers associated with dendritic cells in addition to CD11c, including CD4. Both CD26 and CD1c were expressed, previously described as markers of distinct DC subsets, together with low levels of BDCA3 (Figure 2G).

We further assayed, by qPCR, transcription factors associated with dendritic cell populations. pDC-specific transcription factors E2-2 and IRF8 were absent in the AIRE-enriched cells, while both IRF4 and BATF3 were expressed at low levels (Figure 2H).

To determine their morphology and intracellular organelles, we visualized AIRE-enriched eTACs, pDCs, and cDCs, by electron microscopy. cDCs displayed large numbers of filopodia, associated with their role as phagocytic cells, while the rounded cell surface of eTACs was more similar to pDCs (Figure 2I). However, pDCs on average contained more multivesicular bodies, while eTACs and cDCs showed similar levels of mitochondria and lipid bodies (Supplementary Figure 3A), indicative of a metabolically active state. Further, eTACs displayed a propensity for more open chromatin, required for active gene transcription, which was also evident from reduced DAPI staining (Supplementary Figures 3B,C).

### AIRE Is Not Associated With Tissue Restricted Antigen Expression in the Periphery

**Figure 3:**
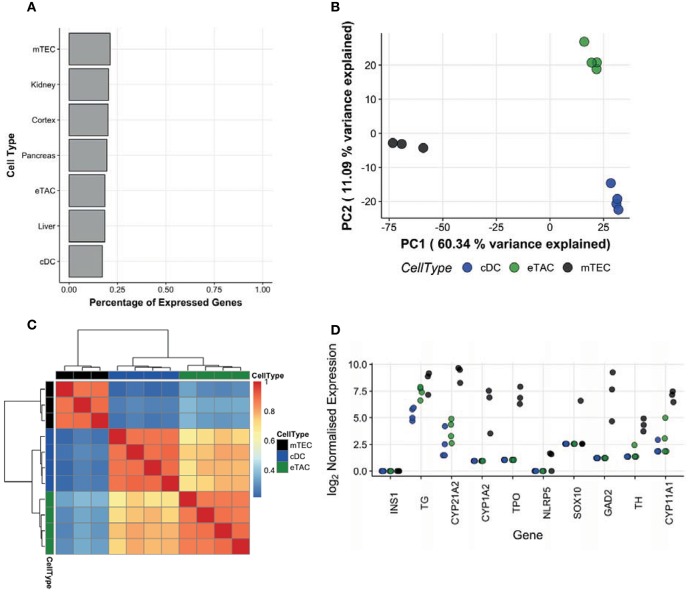
AIRE in peripheral dendritic cells is not associated with tissue-restricted antigen expression. Populations of eTACs (*n* = 4), cDCs (*n* = 4, paired donors) and mTECs (*n* = 3, distinct donors) were sorted and the entire transcriptome sequenced as a bulk population, for detecting tissue-restricted antigen expression. **(A)** Genes were assigned as tissue-restricted using the tissue-specificity index (see Materials and Methods). Proportions of expressed genes in this category in eTACs, cDCs, and mTECs and in specific peripheral tissues are shown. **(B)** Principal components analysis of eTACs, cDCs, and mTECs samples using all expressed TRA genes. Shown are the first 2 major components of variation, corresponding to 60.34 and 11.09% of the variance, respectively. **(C)** A heatmap illustrating the similarity of cDCs, eTACs, and mTECs based on the Pearson correlation of expression of tissue restricted antigen genes. Dendrograms represent average-linkage hierarchical clustering on the Pearson correlation values. **(D)** Log_2_ expression of genes homologous to AIRE-dependent TRAs identified in mice within bulk sequenced cDCs (blue), eTACs (green), and mTECs (black). See also Supplementary Figure 4.

Based on the enrichment of AIRE in these CCR7+ cells and the known role of AIRE in promiscuous expression of tissue-restricted antigen (TRA) genes in mTECs, we examined the transcriptomes of eTACs with respect to TRAs. As TRAs are transcribed stochastically and at low cell frequency (13), we sequenced the bulk transcriptomes of cell populations, rather than single cells, to maximize our ability to capture these transcripts. *AIRE* transcripts were significantly upregulated in sorted CCR7+ eTACs compared to cDCs (6.91 log-fold change, adjusted *p*-value = 2.76 × 10^−4^). We compared the TRA expression in eTACs with that of cDCs from 4 paired tonsil donors, and used postnatal mTECs from 3 unrelated donors as positive controls. Expressed genes were assigned a tissue-restricted status using the tissue-specificity index calculated as per Yanai et al. (14), and as described in materials and methods. A comparison of the proportions of TRAs expressed by each cell population indicated that eTACs do not preferentially express these antigens beyond the extent expected in peripheral tissues such as the pancreas or kidney (Figure 3A), and contrary to mTECs in which enrichment was visible. This pattern of expression was corroborated when we compared the whole distribution of the tissue-specificity index between eTACs, cDCs, and mTECs. We observed a clear and consistent shift toward greater tissue-specific expression in mTECs, indicated by a higher τ-index (see Materials and Methods), which was not mirrored by eTACs (Supplementary Figure 4). By performing principal components analysis (PCA) focused upon TRAs, eTACs had greater transcriptomic similarity to cDCs than to mTECs (Figure 3B), occupying a similar space along principal component 1, which accounted for ~60% of the variation. This was also evident by Pearson correlation of TRA expression, with eTACs and cDCs clustering into a single clade distinct from mTECs (Figure 3C). The exact AIRE-dependence of TRAs in human mTEC has not yet been established. However, using known murine Aire-dependent TRAs (13) we observed the almost complete absence of many homologous TRAs in human eTACs (Figure 3D), whilst others were expressed at comparable levels between cDCs and eTACs; these TRAs were much more abundant in mTECs. Therefore, unlike in the thymus, AIRE expression in peripheral antigen presenting cells identified here was not associated with promiscuous gene expression of TRAs.

### AIRE-Enriched CCR7+CD127+MHCII+ DCs Are Functional DCs With T Cell Stimulatory Potential

**Figure 4:**
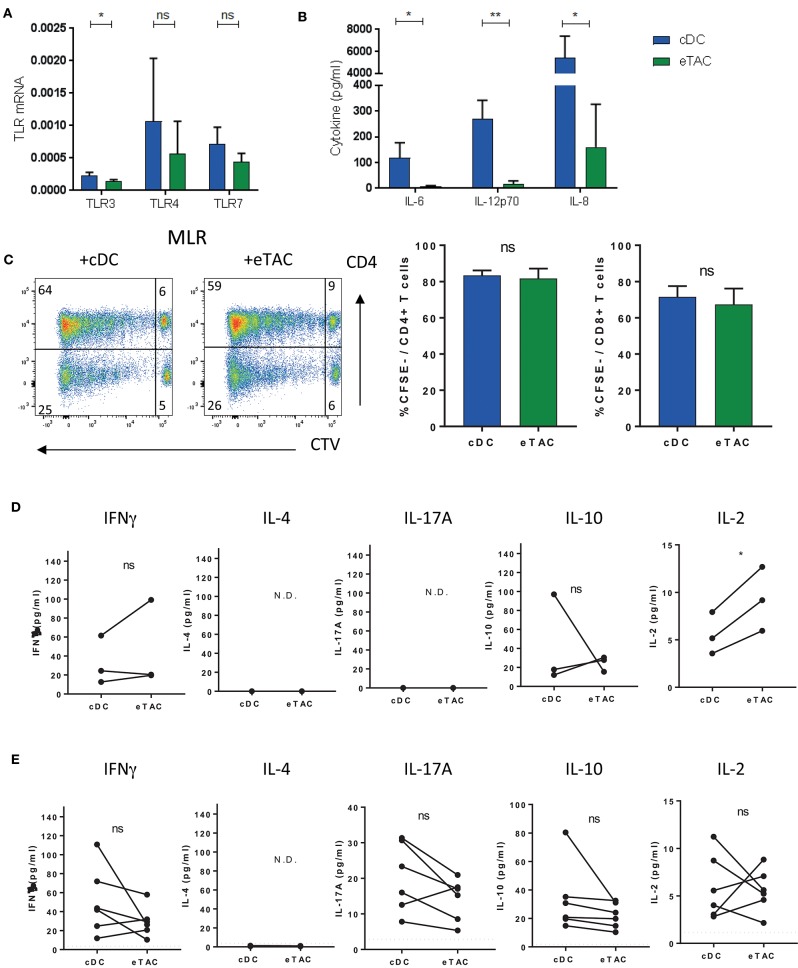
Dendritic cells expressing AIRE can modulate T cell responses **(A)** mRNA expression of select TLRs in cDCs (blue) and eTACs (green) relative to β-actin (*n* = 3) (mean± SD ns = not significant, *p < 0.05 by paired *t*-test) **(B)** cDCs or eTACs were stimulated with a cocktail of TLR stimuli; LPS plus poly(I:C) plus R848. Concentrations (pg/ml) of IL-6, IL12p70, and IL-8 were determined in culture supernatants after 24 h (*n* = 3) (mean ± SD ns = not-significant, *p < 0.05 by paired *t*-test) **(C)** Representative FACS plots of mixed lymphocyte reactions (MLR) of allogeneic CD4+ and CD8+ T cells 6 days after coculture with cDCs or eTACs, proliferation was determined by CTV dilution and cumulative data plotted (*n* = 3) (mean ± SEM, ns = not-significant by paired *t*-test) **(D)** Concentration of IFNγ, IL-4, IL-17A, IL-10, and IL-2 in 6 day culture supernatants from coculture of allogeneic naive CD4+ T cells with cDCs or eTACs (*n* = 3) N.D. indicates cytokines not detected (*p < 0.05, ns = not-significant by paired *t*-test) **(E)** Concentration of IFNγ, IL-4. IL-17A, IL-10, and IL-2 in 6 day culture supernatants from coculture of autologous memory CD4+ T cells with cDCs or eTACs loaded with tetanus toxoid. Dotted lines indicate concentration of each cytokine in the absence of tetanus toxoid, which was <6 pg/ml in all cases (*n* = 6) N.D. indicates cytokines not detected (ns = not-significant by paired *t*-test). See also Supplementary Figure 5.

To assess the functional capacity of eTACs, we assessed expression of selected membrane and endosomal toll-like receptors (TLR) and their stimulatory capacity. While TLR3, 4 and 7 were expressed by eTACs at the RNA level (Figure 4A), stimulation of these TLRs by a cocktail of agonists (LPS, poly(I:C) and R848) induced production of less IL-6, IL12p70, and IL-8 compared to cDCs (Figure 4B). This was also true when cells were non-specifically stimulated with PMA and ionomycin (Supplementary Figure 5A), and was not due to a difference in cell death (Supplementary Figure 5B).

Mixed lymphocyte reactions of either eTACs or cDCs with allogeneic T cells demonstrated the ability of eTACs to stimulate both CD4+ and CD8+ T cells, with robust proliferation of both, comparable with induction levels by cDCs (Figure 4C).

Mouse eTACs have previously been reported to functionally inactivate CD4+ T cells (12). We investigated cytokine production during allogeneic co-culture of eTACs with naïve CD4+ T cells compared to co-culture with cDCs. We did not observe evidence for anergy with similar levels of IFNγ induced by both eTACs and cDCs. Indeed higher levels of IL-2 were produced during co-culture with eTACs. Levels of IL-10 were highly variable, and neither type-2 IL-4 nor type-17 IL-17A could be detected (Figure 4D). For co-cultures with memory CD4+ T cells we utilized memory responses induced by tetanus vaccination to mimic an antigen-specific stimulation. Either eTACs or cDCs were pre-loaded with tetanus toxoid before the addition of autologous memory CD4+ T cells, containing vaccine-induced tetanus-specific memory responses. Following a 6-day co-culture, supernatants were assessed for cytokine responses (Figure 4E). While IL-4 was not detected in either co-culture, IFNγ, IL-17A, IL-2, and IL-10 were induced to a similar level by T cells interacting with either eTACs or cDCs, demonstrating the ability of eTACs to present exogenous antigen and induce functional responses in CD4+ T cells. We further tested the ability of tetanus-specific memory CD4+ T cells to respond to re-stimulation following interaction with eTACs. Following co-culture with either tetanus toxoid-loaded eTACs or cDCs, CD4+ T cells were re-sorted and stimulated with antigen-bearing cDCs. After a further 4 day co-culture, CD4+ T cells were assessed for functional capability by IFNγ production following PMA + ionomcyin stimulation. We did not observe any difference in the response of these memory CD4+ T cells following interaction with either eTACs or cDCs (Supplementary Figure 5C). Thus, we can conclude that eTACs are functionally equivalent to cDCs in their capacity to stimulate both CD4+ naïve and memory responses, and do not induce an anergic phenotype.

### AIRE Is Expressed During DC Maturation

**Figure 5:**
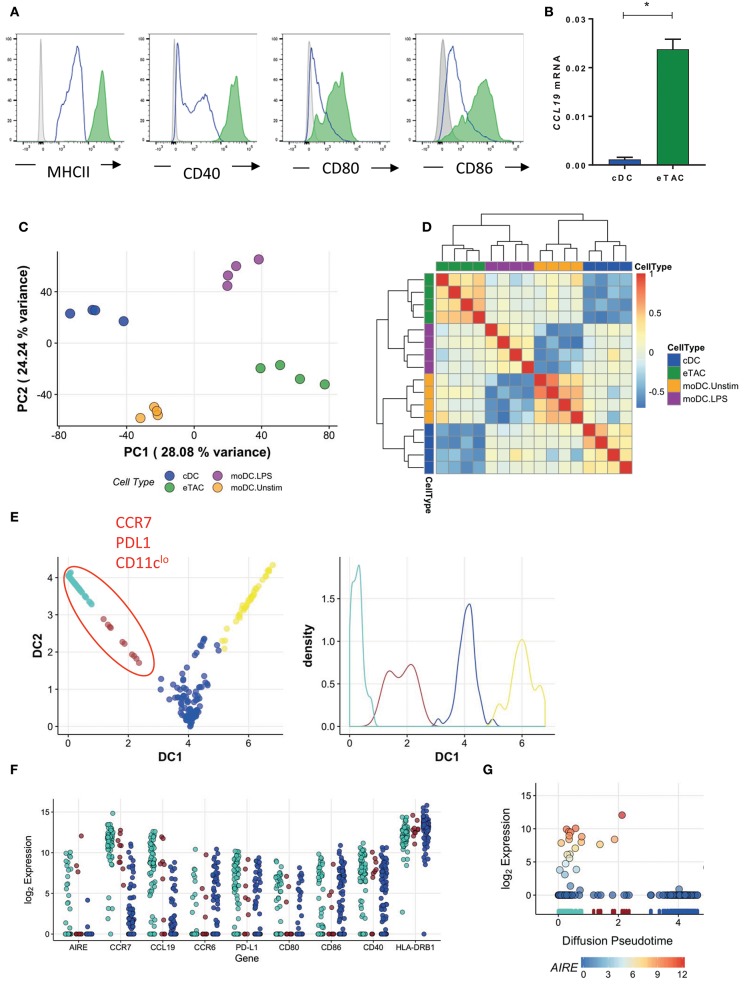
AIRE is upregulated in mature dendritic cells **(A)** Flow cytometric analysis of antigen presenting and costimulatory molecules by tonsil eTACs (filled green) and cDCs (blue line) compared to isotype (gray) **(B)** CCL19 mRNA expression in cDCs and eTACs (*n* = 3), normalized to b-actin levels (mean ± SD, *p < 0.05 by paired *t*-test) **(C)** PCA analysis of the transcriptome of eTACs (green) and cDCs (blue) compared to unstimulated (orange; moDC Unstim) and LPS stimulated (purple; moDC LPS) monocyte-derived DCs (Zaal et al.). The plotted axes are principal component 1 (PC1) and 2 (PC2) which together explain ~50% of the variation. The first (x) axis separates the stimulation profile. **(D)** Heatmap shows Pearson correlation values of 12 principal components, which capture >95% of the total variation for eTACs, cDCs, unstimulated, and LPS-stimulated moDCs **(E)** Pseudotime analysis of single cells showing CCR7+ PDL1+ CD11clo cells on one trajectory, plotting cells according to diffusion component (DC) 1 and 2, left. Right shows cell density along each branching trajectory, and colored accordingly **(F)** Gene expression of select genes in the blue, brown, and turquoise meta-stable states along the first diffusion component **(G)** Log_2_ AIRE gene expression over the first diffusion component, which represents the inferred pseudotime. The RUG plot below indicates which meta-stable state cells are assigned to, i.e., turquoise, brown, blue, and yellow, as identified in E. Points are colored by log_2_ *AIRE* expression for emphasis. See also Supplementary Figure 6.

The surface phenotype of eTACs was highly similar to that of mature DCs; in addition to expression of CCR7 and PDL1, eTACs within both tonsils (Figure 5A) and thymus (Supplementary Figure 2) displayed high levels of MHCII and costimulatory molecules including CD40, CD80, and CD86. Furthermore, eTACs showed specific expression of CCL19 mRNA (Figure 5B), a T cell chemoattractant associated with DC maturation (15, 16).

To determine whether eTACs represent activated and mature DCs, we compared the transcriptomes of eTACs and cDCs with those of unstimulated and LPS-stimulated monocyte-derived DCs (designated moDC), from Zaal et al. (17). After controlling for experimental variation using surrogate variable analysis, we compared these DCs to our tonsil-derived cDCs and eTACs in a reduced dimensional space defined by PCA (Figure 5C). As expected, cDCs were transcriptionally more similar to unstimulated moDCs while eTACs were more similar to the LPS-stimulated moDCs along the first major axis of variation. These relationships were maintained in higher dimensions, illustrated by the high correlations, and co-clustering, between eTACs and the LPS-stimulated moDCs (Figure 5D).

To determine when during the maturation process *AIRE* might be expressed, we constructed a maturation trajectory from our single-cell RNA-sequencing data of the wider EpCAM+ sorted cells. Using a diffusion map, which defines vectors through the high dimensional transcriptome space via a diffusion process, cells were ordered according to the transition probability of progressing from one cell to another. Thus, this trajectory represents the underlying biological progression from one cellular state to another. While analysis of the single-cell RNA-sequencing data had demonstrated that *AIRE* transcripts were enriched within CCR7+CD127+ cells, transcripts were still expressed in only a proportion of cells (23/151 cells; 15.2%) (Figure 1F). The inferred pseudotime trajectory revealed two major branches (Figure 5E) from which we identified four groups of cells as regions of greatest cellular density using a K-means partitioning along pseudotime (Figure 5E). The yellow branch of cells appeared to represent monocytic cells, with significant upregulation of genes such as *CD14* and *MMP9*, but not AIRE (Supplementary Figure 6A) and excluded from the refined phenotype of eTACs by addition of CD14 in the lineage cocktail. Therefore, we did not consider these further in our maturation trajectory analysis.

On the other hand*, AIRE* expression was enriched in the turquoise group of cells (Figure 5F) together with upregulated expression of CCR7 and CCL19 (Figure 5F, Supplementary Figures 6B,C), as observed previously. These markers were also relatively enriched in the brown group of cells, while *CCR6*–a chemokine receptor expressed in DCs prior to *CCR7* and downregulated upon migration to, and maturation within, secondary lymphoid organs–was relatively downregulated (Figure 5F). This suggests a development trajectory in which DC maturation progresses from the brown (more immature) to turquoise (more mature) group. Interestingly, AIRE expression was concentrated toward the terminal end of the pseudotime branch (turquoise cells) (Figure 5G), indicating that AIRE expression is associated with a more mature phenotype. Further, as indicated in Figure 5G, levels of AIRE vary across this small number of cells, which indicates that eTACs do not represent a stable sub-population of cells and is suggestive instead of transient expression. Together, this would imply that eTACs represent a temporal state in the maturation of DC migrating to secondary lymphoid organs, such as lymph nodes and tonsils, and during which *AIRE* may be transiently expressed.

## Discussion

The existence of a population of *AIRE* expressing cells outside of the thymus has long been suggested, even from the first identification of the *AIRE* gene (3). However, the identity of this cell population—particularly in humans—has been elusive. Here we identify *AIRE* expressing cells in human tonsils. Our experimental investigations revealed *AIRE* to be expressed by mature DC, characterized by high levels of antigen presentation with reduced TLR responsiveness, and expression of both CCR7 and PDL1. By single cell sequencing analysis we further observed *AIRE* to be transiently expressed by this population during their differentiation to a mature phenotype. Importantly, and somewhat surprisingly, we found expression of AIRE in the absence of enrichment in promiscuously-expressed TRAs. The transient nature of *AIRE* expression, together with the lack of TRAs, provides some explanation for the difficulty in characterizing human eTACs to date.

eTACs were originally described in mice, with the aid of an *Aire*-GFP reporter, yet even here their identification has not been straightforward. eTACs in mice were first described as antigen-presenting, non-hematopoietic, stromal cells (7), but later re-defined as hematopoietic with low expression levels of CD45 (12). This low CD45 expression is thought to explain the prior identification of *AIRE* in stromal populations, which could be further confused by the expression of EpCAM, a marker of eTACs in mice (12). While human AIRE+ cells were also CD45^lo^ and appeared to express EpCAM on the cell surface, we did not detect transcripts for EpCAM in human *AIRE*+ cells of either tonsil or thymus. Detection of EpCAM on these cells by antibody-staining, here and in mice (12), may reflect acquisition of EpCAM by a form of transfer, for example by exosome uptake or membrane exchange. Indeed, mature DC within the thymus have previously been described to acquire EpCAM, together with other molecules, from neighboring thymic epithelial cells (18, 19).

Instead, we found CCR7 to be the most specifically and strongly up-regulated cell surface marker in *AIRE*-enriched cells, at both the transcript and protein level. Sorting by this marker lead to a much more profound enrichment in *AIRE* than sorting by EpCAM. Further phenotyping demonstrated that these cells express several DC markers in addition to CD11c, including surface markers and transcription factors. This description is supported by the immunohistochemical analyses of human secondary lymph nodes, including tonsils, and gut-associated lymphoid tissue by Poliani et al. (9) who found that AIRE co-localized with HLA-DR and CD40, myeloid markers such as CD11c, and, importantly, with CCR7. In addition, these CCR7+CD127+MHCII+ cells displayed T cell stimulatory capacity *ex vivo* in our experiments which, together with their phenotype, led to their identification as a population of DCs.

Expression of CCR7 by DCs is associated with a migratory and mature phenotype. Up-regulated expression of CCR7 and down-regulation of tissue-homing receptors such as CCR6 are required for migration and positioning of DC within secondary lymphoid organs (20, 21). The *AIRE*-enriched cells identified here also expressed the receptor for IL-7, CD127, which was absent from cDCs. CD127 has previously been described to be specifically expressed by migratory DC within mice, and thought to be important for their positioning near IL-7 producing reticular cells of the T cell zone (22); indeed we identified AIRE+ cells within the T cell zones of the tonsils. Upon activation, DCs also up-regulate MHCII and costimulatory molecules CD80 and CD86, and down-regulate CD11c (20). This maturation process is in line with a switch in functional priorities from antigen capture to antigen presentation. These findings are consistent with the CD11c^lo^MHCII^hi^CD80^hi^CD86^hi^ phenotype, reduced TLR responsiveness and pro-inflammatory cytokine production, and the more rounded, less dendritic morphology of eTACs. While this phenotype may result from the inflammatory environment from which these cells were isolated (the indication for adenectomy being due to recurrent adenitis), we also identified AIRE-expressing cells within the postnatal thymus, under physiological steady state conditions, and, importantly, these cells had the same phenotype. This included the specific expression of CCR7, CD127, and PDL1. Together these results suggest this phenotype is not simply a result of inflammation, and may be indicative of a homeostatic maturation and/or migration of DCs, with a fraction of DCs previously described to undergo constitutive maturation (23). Interestingly TSLP, a cytokine that also interacts with CD127, has been described to activate thymic CD11c+CD11b- DCs. When treated with TSLP these thymic DCs could stimulate Treg cells from CD4+ thymocytes but not from peripheral CD4+ T cells (24). Although not directly analyzed, the ability to responsd to TSLP would suggest expression of CD127 by these thymic DC, and therefore it is tempting to speculate that the CCR7+ CD127+ AIRE+ DC that we identified in the thymus may be identical to these TSLP-responsive DC.

While mature DCs within tonsils were enriched in *AIRE* expression there was, however, a wide variation between donors, and single cell sequencing revealed *AIRE* to be expressed by only a proportion of cells and at varying levels. Immunohistochemical analyses, both here and in previous work (9, 12), identifed AIRE+ cells at a low frequency within secondary lymphoid organs in humans. Furthermore, our pseudotime inference from the single cell transcriptomes suggests that AIRE+ cells do not represent a single stable population. This implies that *AIRE* expression does not correspond to a distinct cell subset within tonsils, but rather an intermediate cellular stage. The increased AIRE expression together with increased CCR7 and PDL1, and decreasing CCR6, expression would indicate that this state is associated with the process of maturation. This is not without precedent, as thymic B cells have been described to induce AIRE expression upon signaling through CD40, with a concomitant up-regulation of MHCII and CD80 (25). This raises the possibility that AIRE expression may also be induced by CD40-mediated NF-κB signaling in DCs, or potentially in only a subset of DCs resident in peripheral lymphoid tissue. The prototypical AIRE expressing cells in the thymus are the medullary epithelial cells (mTECs). Amongst mTECs, AIRE expression is restricted to those bearing a mature phenotype, a differentiation state defined by high expression of MHCII and CD80 (1, 26) as well as expression of PDL1 (27, 28). Therefore, the MHCII^hi^, CD80^hi^, PDL1+ phenotype of AIRE-expressing DCs identified here by and large mirrors that of AIRE-expressing mTECs. Furthermore, mTECs are known to secrete CCL19 to attract immature thymocytes for negative selection (29); CCL19 mRNA was specifically enriched within the AIRE+ mature DCs. Furthermore, in mice, typically only 50–60% of phenotypically mature mTECs express AIRE at any one time (30). Moreoever, AIRE expression is also restricted to a particular stage during mTEC differentiation from immature epithelia to a post-mitotic, terminally differentiated cell stage (26, 31).

Our observations raise the question of what role AIRE plays within these DCs. Expression of AIRE during a maturation process, rather than by a distinct subset, would imply a distinct role for the AIRE gene during this process as opposed to a distinct function of AIRE-expressing cells, and indeed we found AIRE-enriched DC to possess the same T cell stimulatory capacity as cDC without inactivation of CD4+ T cells as had been observed previously (12). The assumption to-date has largely been that AIRE is associated with expression of TRAs. While this may be true within the thymus, it may not necessarily be true for peripheral cells. Indeed, while identification of the expression of AIRE-controlled TRAs in the thymus has been reproduced across studies, reports within the periphery have been less consistent (32). We found no evidence for an enriched expression of TRA, or human homologs of known mouse Aire-dependent TRAs, within human AIRE+ DCs. This would suggest that AIRE might perform an alternative role within these cells. Indeed, roles for AIRE in other cellular processes have been described. For example, AIRE has been reported to promote the expression of chemokines including CCL19 (33, 34)–a chemokine which we found enriched in these cells. A central role for AIRE in controlling the differentiation program of mTECs toward terminal differentiation has also been reported (31). Likewise, AIRE may be directly involved in the differentiation process of DCs during maturation. Overexpression of AIRE in a monocytic cell line induced changes consistent with the maturation process of monocyte-derived DCs (35). Further, monocyte-derived DCs of APECED patients, and thus lacking functional AIRE, are functionally impaired (36). Indeed, not all symptoms in these patients can be explained by a loss of tolerance, such as an increased susceptibility to fungal infections. Therefore, identification of human “eTACs” as maturing DCs signifies an important step not only in understanding DC biology, but in further understanding autoimmune and other diseases such as APECED.

## Materials and Methods

### Human Tissues

All tissues were used with approval of the Medical Ethical Committee of the Academic Medical Center, Amsterdam according to the Research Code of the Academic Medical Center of the University of Amsterdam. Tonsils were obtained from pediatric and adult tonsillectomies. Postnatal thymi were obtained from surgical specimens removed from children up to 3 years of age undergoing open heart surgery. Tissues were disrupted by mechanical means and pressed through a stainless steel mesh to obtain a single-cell suspension. Mononuclear cells were isolated from a Ficoll–Hypaque density gradient (Lymphoprep; Axis-Shield).

### Cell Isolation

Tonsil mononuclear cells were depleted of T and B cells by FITC-conjugated anti-CD3 (OKT3; BioLegend) and anti-CD19 (HIB19; BioLegend) and anti- FITC microbeads (Miltenyi Biotec) and passed over an LD magnetic column (Miltenyi Biotec). Cells were subsequently sorted to purity using a FACSAria (BD Biosciences) and antibodies against lineage (CD3, CD19, CD20, CD94, CD34, CD16, CD14, TCRαβ, TCRγδ), HLA-DR, CD11c, CCR7, CD123, and BDCA2. eTACs were sorted as Lin-CD123-BDCA2-HLADR+CCR7+, cDCs as Lin-CD123-BDCA2-HLADR+CD11c+ and pDCs as Lin-HLADR+CD123+BDCA2+. Cells were incubated with an FcR block (Miltenyi Biotec) prior to antibody staining.

T cells were FACS sorted directly from mononuclear cells using antibodies against CD4, CD8, CD45RO, and CD25. For mixed lymphocyte reactions equal numbers of CD4+CD8-CD25- and CD4-CD8+CD25- cells were sorted. Memory CD4+ T cells were sorted as CD4+CD8-CD45RO+CD25-. Staining of CD3 was omitted to prevent potential cross-linking and signaling via CD3.

For isolation of mTEC postnatal thymic tissue was cut into small pieces and digested for 1 h at 37°C with IMDM (GIBCO) containing Liberase TM (125 μg/ml). The resulting cell suspension was passed through a 70 μm cell strainer and then epithelial cells selected using anti-EpCAM microbeads (Miltenyi Biotec) as per the manufacturer's recommendations. mTECs were further selected from the positive fraction as CD45^−^CD80^+^HLA-DR^+^EpCAM^+^ cells by flow cytometric cell sorting.

### Antibodies

Antibodies were used as detailed in Supplementary Table S1. Dead cell discrimination was performed using live/dead fixable green dead cell stain kit (Life Technologies). Data was analyzed with FlowJo software version 10 (FlowJo, LLC).

### CyTOF

Directly conjugated antibodies were purchased from Fluidigm, or MaxPar-ready antibodies purchased from BioLegend and conjugated following Fluidigm's Lanthanide Labeling of Antibodies Pre-Load method with X8 polymers. Conjugated antibodies were stored in Antibody Stabilizer (Candor Biosciences). Antibodies used are shown in Supplementary Table S2.

First, tonsil cells were depleted of T (CD3) and B (CD19) cells as previously, and left overnight at 4 degrees. The next day, cells from 2 donors were pooled and resuspended in a Cell-ID Cisplatin viability stain (Fluidigm) at 5 μM for 5 min at room temperature. Fc receptors were then blocked by a 10 min incubation at room temperature with FcR block (BioLegend) before addition of surface antibodies for a further 30 min. Cells were washed twice with staining buffer (PBS + 1% BSA) and incubated overnight in 250 nm of Cell-ID intercalator (Fluidigm) in 2% paraformaldehyde (Pierce). Before acquiring cells were washed extensively in staining buffer, PBS and distilled water, and EQ Four Element Calibration beads added (Fluidigm). CyTOF data were acquired on-the-fly, using dual-count mode and noise-reduction on. All other settings were either default settings or optimized with tuning solution, as instructed by Fluidigm. After data acquisition, the mass bead signal was used to normalize the short-term signal fluctuations. Analyses were performed with Cytobank (37).

### Co-cultures

All cells were cultured in Yssel's medium supplemented with 3% (vol/vol) heat inactivated normal human AB serum. eTAC or cDC were cocultured with T cells at a 1:2 ratio. In specific experiments T cells were stained with Cell Trace Violet (Thermo Fisher Scientific) at a concentration of 5 μM before coculture. In antigen-specific experiments, eTAC or cDC were first incubated with 10 μg/ml of tetanus toxoid (Calbiochem) before addition of autologous memory CD4+ T cells. All cocultures were performed for 6–7 days.

### Cytokine Measurements

For detection of intracellular cytokines, cells after coculture were activated with PMA (50 ng/ml; Sigma) and ionomycin (1 μM;Merk) for 6 h in the presence of GolgiPlug (1 μl/ml BD Biosciences) for the final 4 h. Cytokines in coculture supernatants were analyzed by LEGENDplex Th cytokine panel (BioLegend).

For activation of eTAC, cDC and pDC, purified subsets were cultured at 5 × 103 cells per well in the presence of LPS (100 ng/ml; Sigma), poly(I:C) (25 μg/ml;Sigma) and R848 (25 μg/ml; Invivogen), or with PMA (50 ng/ml; Sigma) and ionomycin (1 μM;Merk). Culture supernatants were harvested after 24 h and analyzed by a custom LEGENDPlex human inflammation panel (BioLegend).

### Quantitative Real-Time PCR

Total RNA was isolated from sort-purified cells with NucleoSpin RNA XS kit (Macherey-Nagel) according to the manufacturer's protocol. Complementary DNA was synthesized with the High-Capacity cDNA reverse transcription kit (Applied Biosystems). Quantitative real-time PCR was performed on a C1000 Thermal cycler CFX96 real-time system (Biorad) with IQ SYBR Green supermix (Bio-Rad). Primers sets used were:

β-actin forward 5′-CACCATTGGCAATGAGCGGTTC-3′, reverse 5′-AGGTCTTTGCGGATGTCCACGT-3′ ; AIRE forward 5′-GAGAGTGCTGAGAAGGACA-3′, reverse 5′-GTTTAATTTCCAGGCACATGA-3′ ;
E2-2 forward 5′-GCCTCTTCACAGTAGTGCCAvTG-3′, reverse 5′-GCTGGTTTGGAGGAAGGATAGC-3′ ;
IRF4 forward 5′-TGATCGACCAGATCGACAGC-3′, reverse 5′-GGGGCACAAGCATAAAAGGT-3′ ;
IRF8 forward 5′-TGGGGATGATCAAAAGGAGCC-3′, reverse 5′-AACTGGCTGGTGTCGAAGAC-3′ ;
BATF3 forward 5′-GAGCCCTGAGGATGATGACAG-3′, reverse 5′-CTTCCCGATCTCTCTCCGCA-3′;
CCL19 forward 5′-CCTCAGCCTGCTGGTTCTCT-3′, reverse 5′-CAGCAGTCTTCAGCATCATTGG-3′;
TLR3 forward 5′-TTGCCTTGTATCTACTTTTGGGG-3′, reverse 5′-TCAACACTGTTATGTTTGTGGGT-3′;
TLR4 forward 5′- AGACCTGTCCCTGAACCCTAT-3′, reverse 5′- CGATGGACTTCTAAACCAGCCA-3′;
TLR7 forward 5′-TCCTTGGGGCTAGATGGTTTC-3′, reverse 5′-TCCACGATCACATGGTTCTTTG-3′;
TLR9 forward 5′-CTGCCACATGACCATCGAG-3′, reverse 5′-GGACAGGGATATGAGGGATTTGG-3′;
EpCAM forward 5′-TTTGCGGACTGCACTTCAGA-3′, reverse 5′-AAGATGTCTTCGTCCCACGC-3′.

### Immunostaining

Cryopreserved tonsil sections were thawed and fixed with acetone for 10 min. Subsequently, slides were washed 3 times with PBS and primary antibody (anti-AIRE: D17 and anti-CD19: HIB19 Santa Cruz) was incubated overnight in 1% (w/v) bovine serum albumin (BSA) in PBS at 4°C. After, slides were washed 3 times with PBS and secondary antibody (Alexa Fluor 594 donkey anti-goat: A11058 and Alexa Fluor 488 donkey anti-mouse: A21202, Invitrogen) was diluted in 1% BSA, 10% (v/v) donkey serum (Jackson Immuno Research Inc.: 017-000-121) in PBS. Incubation with secondary antibody was 1 h at room temperature after which the slides were washed 3 times with PBS. Finally, the slides were mounted with ProLong Gold Antifade Mountant (Invitrogen). Stained slides were imaged using a Leica TCS SP8 X Confocal Microscope (Leica, Wetzlar, Germany). Images were captured by using Leica software.

### Electron Microscopy

Directly after sort purification cells were fixed in 1% glutaraldehyde and 4% PFA in 0.1 M sodiumcacodylate buffer (McDowell fixative) and post-fixed with 1% osmiumtetroxide (OsO4, Electronmicroscopy sciences, Hatfield, PA, USA) in water. Subsequently, the samples were dehydrated in an alcohol series and embedded into Epon (LX-112 resin Ladd research, Williston, VT, USA). Ultrathin epon sections were collected on formvar-coated grids, counterstained with uranyl acetate and lead citrate and visualized with a transmission electron microscope (FEI Tecnai 12). Images were taken from each subset and organelles quantified in a blinded manner.

### Single-Cell RNA Sequencing

CD45+HLADR+CD127+CD40+EpCAM+ cells were sorted as single cells into wells of a 384 well-containing lysis buffer and ERCC92 RNA at a dilution of 1:1,000,000 of mix 1 per well (ThermoFisher). Single cell cDNA was generated using the SMARTSeq2 protocol (38), and multiplexed libraries for high throughput sequencing were generated using the Nextera XT preparation (Illumina). Multiplexed libraries were sequenced on a HiSeq 2500 to generate 150 bp paired end (PE) reads.

### Single Cell RNA Sequencing Processing

Defined contaminating sequencing and library preparation adaptors were removed from single cell RNA-seq reads using trimmomatic *(-a AGATCGGAAGAGC –overlap 18 –minimum-length 25 –maximum-length 28 -q 20*) (39). Trimmed reads were aligned to the *Homo sapiens* genome (hg38) concatenated with the ERCC92 sequences using STAR v4.2 (40), requiring ≥95% of bases to align perfectly to the reference genome *(–outFilterType BySJout –outSAMattributes All*). Single cell alignments were deduplicated using Picard Tools *MarkDuplicates* (http://broadinstitute.github.io/picard/), prior to quantification of protein-coding gene expression using featureCounts (41), summarized at the gene level using the *Homo sapiens* Ensembl build 86 as a reference (42).

### Single Cell RNA Sequencing Quality Control

The quality of single cells was assessed based on the total number of reads, the number of genes detected per cell as well as the percentage of expression attributable to the ERCC spike-in genes. Poor quality cells were removed on the basis of the following filters; total unique reads <100 k, < 1000 genes expressed and sparsity >95% (number of 0-counts across all genes). In total 217 cells passed quality control measures and were used for further analysis.

### Normalization of Single Cell Gene Expression and Definition of Highly Variable Genes

Genes that displayed high sparsity of expression across cells (>95% 0-counts) were removed, and cell-specific size factors were estimated using the deconvolution method (43). Gene expression counts were normalized by these size factors prior to a log_2_ transformation, implemented in the Bioconductor package *scran* (43). Highly variable genes (HVGs) were defined across all 217 cells by fitting the squared coefficient of variation (CV^2^) to the mean log_2_ expression, parameterized as in Brennecke et al. (44). Genes were tested for excessive deviation from the expected trend using a χ^2^ test. Genes with a *p*-value ≤ 0.01 were designated as highly variable, resulting in 128 HVGs.

### Dimensionality Reduction and Visualization of Single Cells

High dimensional single cell transcriptomes were visualized using dimensionality reduction techniques. Principal components analysis, using the centered and scaled log_2_ expression values, was used to visualize the major axes of variation through the single cell transcriptome data. The high dimensional embedding of cells into 2 dimensions was performed using *t*-distributed stochastic neighbor embedding (tSNE) (45), with the 128 HVGs and a perplexity value set to 50, implemented in the R package *Rtsne*.

### Single Cell Clustering

Assignment of single cells to discrete clusters was performed by first calculating the Spearman correlation dissimilarity (1–ρ), which was used as input to hierarchical clustering using the average linkage algorithm. Clusters were defined using the dynamic tree cutting algorithm (minimum module size 30 cells), implemented in the Bioconductor package *WGCNA* (46). Cell similarities were visualized in heatmaps using the *pheatmap* package in R (47).

### Single Cell Pseudotime Inference

The maturation trajectory of single cells was inferred from the diffusion map constructed on the HVGs defined above. Specifically, the first 3 eigenvalues from the cosine normalized gene expression matrix were used to construct the diffusion map across cells (*k* = 21), implemented in the Bioconductor package *destiny* (48, 49). Meta-stable states along the inferred pseudotime were assigned using a k-means partitioning of the first diffusion component into 4 clusters, implemented in R (47).

### Single Cell Differential Expression Testing

Differentially expressed genes were tested between defined groups (e.g., clusters, meta-stable states, etc. ) by fitting a generalized linear model to the average log_2_ expression values, blocking on potential nuisance factors, cell plate position (by column) and donor. Empirical Bayes shrinkage of the log fold change estimates was performed prior to formal differential expression testing against a null hypothesis of no difference in log_2_ fold changes by a moderated *t*-test, implemented in the Biconductor package *limma* (50). Differentially expressed genes were defined based on a false discovery rate (FDR) of 1% for each analysis.

### Tissue Restricted Antigen Definition

Cap analysis of gene expression (CAGE) with high throughout sequencing (CAGE-seq) run length normalized (RLE) tag expression data were downloaded from the FANTOM5 consortium website (URL). The human samples were manually placed into 107 tissue groups. Tissue specificity of gene expression was calculated across these 107 tissue groups using the τ-index as per (14). Specifically the τ-index is calculated per gene as follows:

$$\begin{array}{l} \tau =\frac{\sum_{i=1}^{N}\left(1-x_{i}\right)}{N-1} \end{array}$$

Where x_i_ is the expression of gene x in tissue *i* ∈{1, 2, …*N*}. Genes with a τ-index value ≥ 0.8 were assigned tissue restricted antigen (TRA) genes, whilst those with a τ-index ≤ 0.4 were assigned to the constitutively expressed group. All other genes were placed into the *Miscellaneous* category.

### Bulk RNA Sequencing

CD45+Lin-HLADR+CD11c+ cDC and CD45+Lin-HLADR+CCR7+ eTAC were sorted from the tonsils of 3 donors directly into Trizol Reagant. Likewise, mTEC were sorted as CD45-HLADR+CD80+EpCAM+ cells from 3 separate donors. Total RNA was extracted using TRIzol reagent (15596-018, Ambion life technologies) according to the manufactures protocol. Briefly, 0.2 × volumes of chloroform (Chloroform stab./Amylene, Biosolve) was added to the homogenate and the tube(s) were shaken vigorously. The tube(s) were incubated for 2–3 min at room temperature and centrifuged (Hettich, rotanta 46 RS) for 1 h (4120 RCF, 4°C). Approximately 70% of the upper aqueous phase was transferred to a clean 15 mL tube with the addition of 1 μl of glycogen (20 μg/μL) (10814-010, Invitrogen) and 0.5 ×volume of isopropanol (33539, Sigma-Aldrich, ) was added. The tube(s) were incubated overnight at −20°C and centrifuged for 30 min (4120 RCF, 4°C). The supernatant was removed and the pellet was washed twice with 80% ethanol (32221-2.5L, Sigma-Aldrich). The total RNA pellet was air-dried for 8 min and dissolved in an appropriate volume of nuclease free water (AM9937, Ambion life technologies) and quantified using the Nanodrop UV-VIS Spectrophotometer (Thermo Fisher Scientific Inc.). Quality and quantity of the total RNA was assessed by the 2100 Bioanalyzer using a Pico chip (Agilent, Santa Clara, CA).

### TruSeq RNA Access Sample Preparation

Strand-specific libraries were generated using the TruSeq RNA access sample preparation kit according to the manufacturer's instructions (Illumina, Part # 15049525 Rev. B). The FF RNA was fragmented, random primed and reverse transcribed using SuperScript II Reverse Transcriptase (Invitrogen, part # 18064-014) with the addition of Actinomycin D. Second strand synthesis was performed using Polymerase I and RNaseH with replacement of dTTP for dUTP. The generated cDNA fragments were 3' end adenylated and ligated to Illumina sequencing adapters and subsequently amplified by 15 cycles of PCR. The libraries were analyzed on a 2100 BioAnalyzer using a 7500 chip (Agilent, Santa Clara, CA). Capture pools of 200 ng per sample were compiled based on library yield and unique adapter sequence, where after target enrichment of the exome is performed. The captured fragments were amplified by a 2nd amplification of 10 PCR cycles. The captured fragments were assessed on a 2100 BioAnalyzer, diluted and equimolar pooled into a 10 nM multiplex sequencing pool, containing 12 samples per pool.

### Sequencing

The libraries were sequenced SR65 using one sequencing lane per pool on a HiSeq2500 with V4 chemistry (Illumina Inc., San Diego). The reads were aligned against the human transcriptome (hg38) using Tophat2 (Tophat version 2.1.0 / Bowtie version 1.1.0) that allows for exon-exon junctions (51, 52). Tophat was guided using a reference genome as well as a reference transcriptome. The reference transcriptome was created using a gene transfer file (GTF) that was downloaded from Ensembl (version 77). Genecounts were generated using Icount, which is based on HTSeq-count (53). Only reads that mapped uniquely to the transcriptome were used to determine the number of reads per gene. The strandedness of the fragments generated during the library preparation was taken into account for both the alignment and the determination of the genecounts.

### Tissue Restricted Antigen Detection Sensitivity

The aligned reads (BAM file) for each individual sample of mTECs, cDCs, and eTACs were randomly down-sampled to ~20M reads using *samtools* (54). Down-sampled BAM files were used to quantify gene expression of all hg38 Ensembl build 86 protein-coding genes using featureCounts (41).

### Bulk RNA-seq Normalization and Differential Expression Testing

Count matrices (genes X samples) were normalized between samples using size factors as described in Anders et al. (55), prior to a log_2_ transformation using a constant offset of 1. Gene count tables were downloaded through the GTEx portal (https://www.gtexportal.org/home/), and 3 samples were randomly selected from the kidney, pancreas and brain cortex samples. Samples were individually normalized using size factors as described above, prior to a log_2_ transformation using a constant offset of 1.

### Functional Enrichment Testing

Functional enrichment testing was carried out on each gene set using the Bioconductor package *goseq* (56). The Gene Ontology Biological Process definitions were used for testing, corrected for gene length bias in all analyses. Statistically significant enrichments were called after multiple testing correction using the Benjamini and Hochberg method, at a false discovery rate (FDR) of 1% (57).

## Code Availability

All code used to analyse bulk and single-cell RNA-sequencing data, and used to generate figure panels are available at https://github.com/MikeDMorgan/Human_eTAC2018.

## Data Availability

Single cell gene expression data are available through ArrayExpress under accession E-MTAB-7381, and bulk RNA-sequencing gene expression data are available through accession E-MTAB-7383.

## Ethics Statement

This study was carried out in accordance with the recommendations of Research Code of the Academic Medical Center of the University of Amsterdam with written informed consent from all subjects. All subjects gave written informed consent in accordance with the Declaration of Helsinki. The protocol was approved by the Medical Ethical Review Committee.

## Author Contributions

JF designed, performed and analyzed experiments, and wrote the manuscript. MM performed all bioinformatic analyses and wrote the manuscript. MB designed experiments. BH performed experiments. VvU and FK provided essential CyTOF support and experimental design. LH, JPvH and ST performed immunohistochemistry. NvdW and DP performed electron microscopy. MA provided essential guidance and discussion. JM provided bioinformatics supervision. GH provided supervision, discussion and guidance. HS performed overall study supervision and design.

### Conflict of Interest Statement

The authors declare that the research was conducted in the absence of any commercial or financial relationships that could be construed as a potential conflict of interest.

## Supplementary Material

Supplementary Figure 1 Single cell RNA sequencing of tonsil cells reveals AIRE expressing cells. Related to Figure 1. **(A)** Human thymus section stained by immunofluorescence for DAPI (blue) and AIRE (red), scale bar indicating 10 μM is shown **(B)** Gating strategy of putative eTACs from tonsil cells depleted of T (CD3) and B (CD19) cells for sorting as single cells for RNA seq analysis **(C)** Log_10_ total read counts of all cells according to donor (A; green B; purple) **(D)** t-SNE plot of single cells from Figure 1E colored according to donors as in b, above **(E)** Heatmap of the top 75 genes with the strongest influence on separating cluster 1 (blue) and cluster 2 (orange) as shown in Figure 1E and clustered by hierarchical clustering **(F)** MA plot differential expression testing between clusters identifies CCR7 and CD11c as principal markers of these different DC populations. Plotted are the mean log_2_ gene expression values (x-axis) against the log_2_ fold change between clusters. Points below the horizontal are up-regulated in cluster 1 (blue), and those above the horizontal are up-regulated in cluster 2 (orange). Points are colored based on a 1% FDR (red: null hypothesis rejected, gray: failed to reject null hypothesis).

Supplementary Figure 2 AIRE expressing cells with equivalent phenotype, and distinct from mTECs, can also be found within human thymus. Related to Figure 2. **(A)** *AIRE* mRNA expression in tonsillar eTACs sorted as per Figure 2A (CCR7+ “eTAC) or Supplementary Figure 1A (EpCAM+ “eTAC) relative to tonsillar cDCs, normalized to β-actin levels (*n* = 6) **(B)** Gating strategy for the isolation of mTECs from postnatal human thymus (PNT) **(C)** Identification of a CCR7+ population amongst CD45+Lin-MHCII+ in PNT **(D)** Histograms show levels of selected markers in CCR7+ intrathymic AIRE expressing cells (iTACs; filled green) and cDCs (blue line) compared to isotype (gray) **(E)** AIRE mRNA transcript levels in iTACs relative to thymic DCs, and normalized to β-actin **(F)** Histograms indicate levels of antigen presenting and co- stimulatory molecules in iTACs (filled green), thymic DCs (blue line) compared to isotype (gray) **(G)** EpCAM mRNA transcript levels in iTACs and mTECs relative to β-actin (*n* = 3), ND, non-detectable.

Supplementary Figure 3 Morphology. Related to Figure 2. **(A)** Example images and numbers of lipid bodies, multivesicular bodies and mitochondria were counted in cells imaged by electron microscopy, **(B)** the percentage of dense (closed) chromatin was estimated for each cell, **(C)** human tonsil sections stained by immunofluorescence for DAPI (blue) and AIRE (green).

Supplementary Figure 4 Tau-index analysis of TRA enrichment. Related to Figure 3. Kernel density plot of Tau-Index in eTACs (green line), cDCs (blue line) compared to mTECs (black line).

Supplementary Figure 5 T cells stimulated with eTACs are still functional to produce IFNγ. Related to Figure 4. **(A)** cDCs or eTACs were left unstimulated or stimulated with PMA + Ionomycin and concentrations (pg/ml) of IL-6, IL12p70, and IL-8 were determined in culture supernatants after 24 h (*n* = 3) (mean ± SD ns = not significant, ^*^*p* < 0.05 by paired *t*-test) **(B)** Percentages of live cells amongst sorted eTACs (green) or cDCs (blue) after stimulation with TLR stimuli (LPS plus poly(I:C) plus R848), PMA + Ionomycin or left unstimulated for 24 h **(C)** Representative flow cytometry plots of PMA+ionomycin-stimulated memory CD4+ T cells restimulated with autologous tetanus-toxoid loaded cDCs after coculture with either eTACs or cDCs as in Figure 4E. Representative of 3 independent experiments.

Supplementary Figure 6 Pseudotime-dependent gene expression analysis. Related to Figure 5. **(A)** MA-plot of genes differentially expressed between **(A)** yellow and blue, **(B)** brown and blue, and **(C)** turquoise and blue cell partitions from the inferred pseudotime trajectory in Figure 5E. Points in red are statistically significantly expressed (FDR 1%). Genes are labeled with a log_2_ fold change >6.
