# Supplementary material for: Maturing Human CD127+ CCR7+ PDL1+ Dendritic Cells Express AIRE in the Absence of Tissue Restricted Antigens
Source: Front Immunol. 2019 Jan 14;9:2902. doi: 10.3389/fimmu.2018.02902 (PMC6340304; doi:10.3389/fimmu.2018.02902)

# Supplementary Figure 1

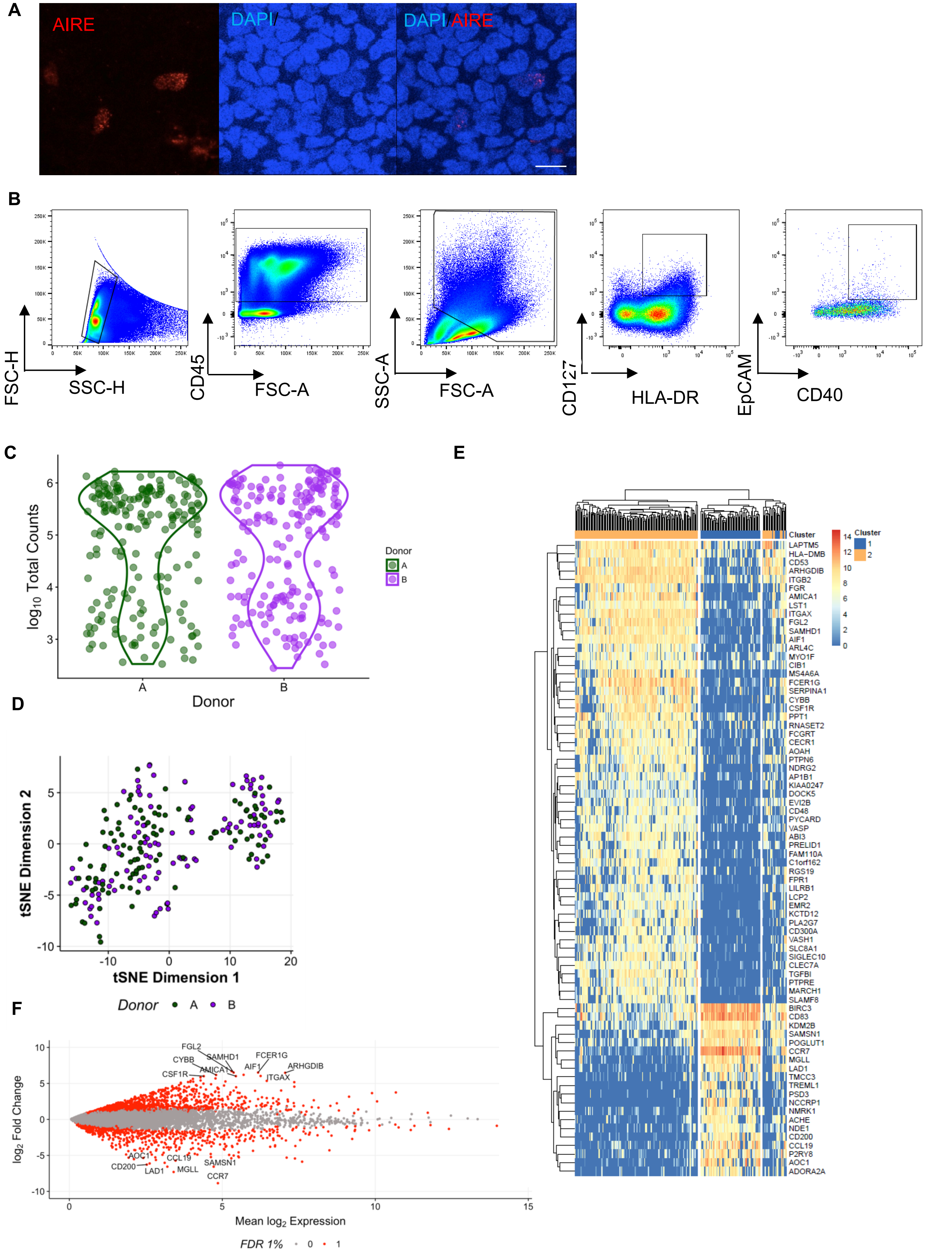

Supplementary Figure 2

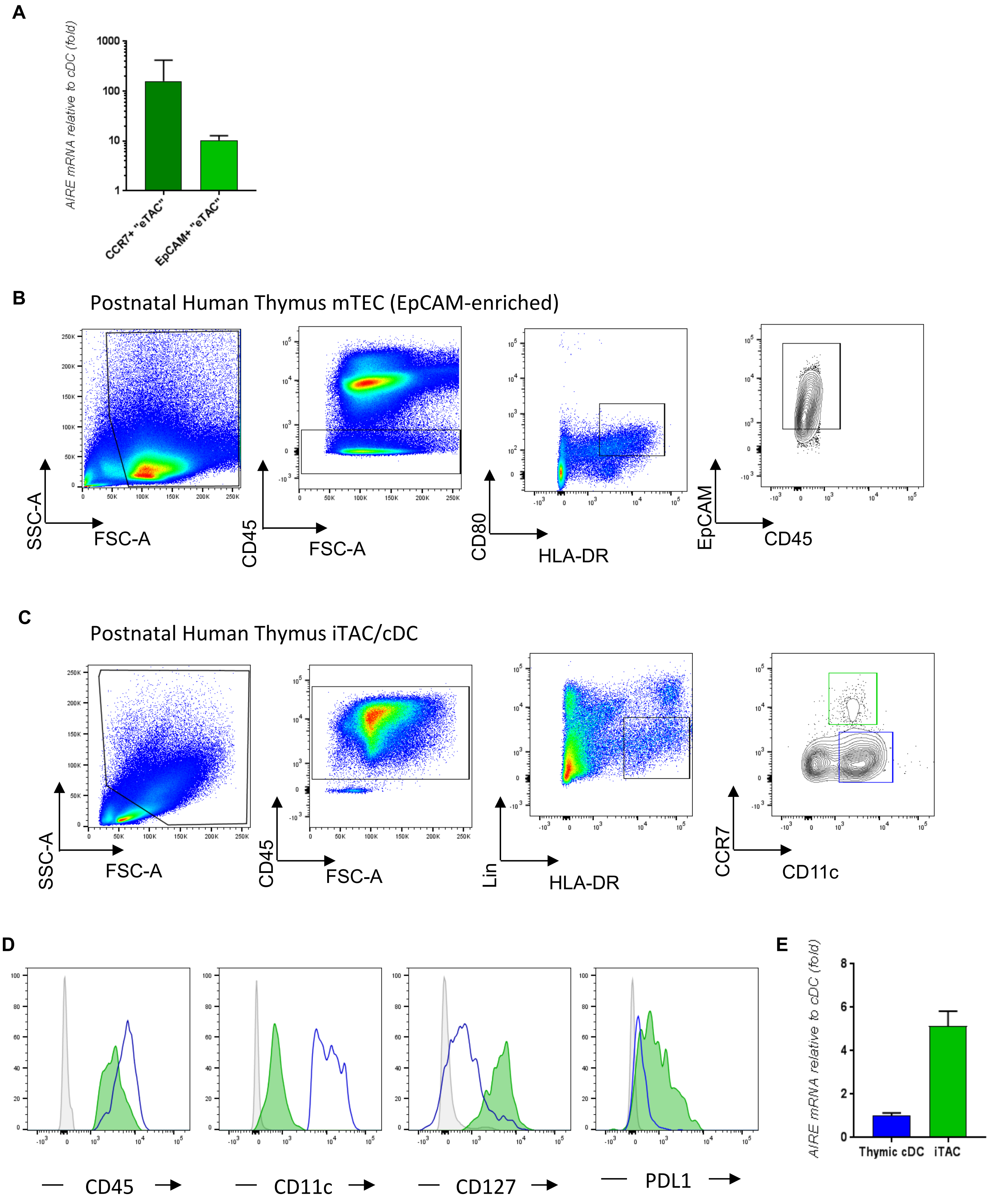

Supplementary Figure 2

F

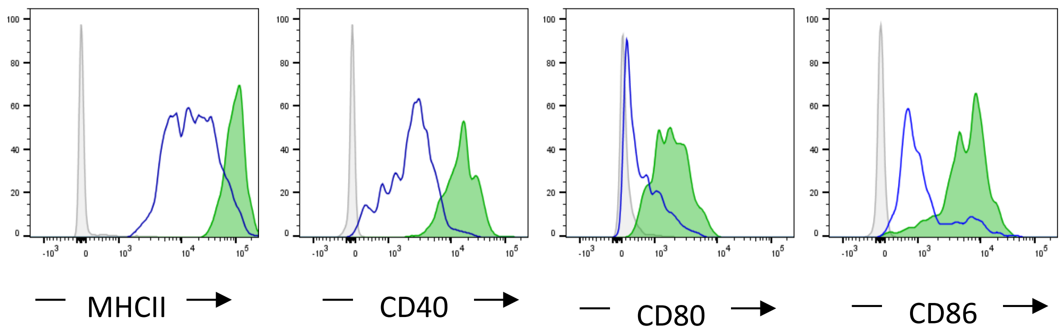


G

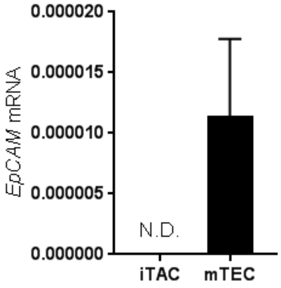

Supplementary Figure 3

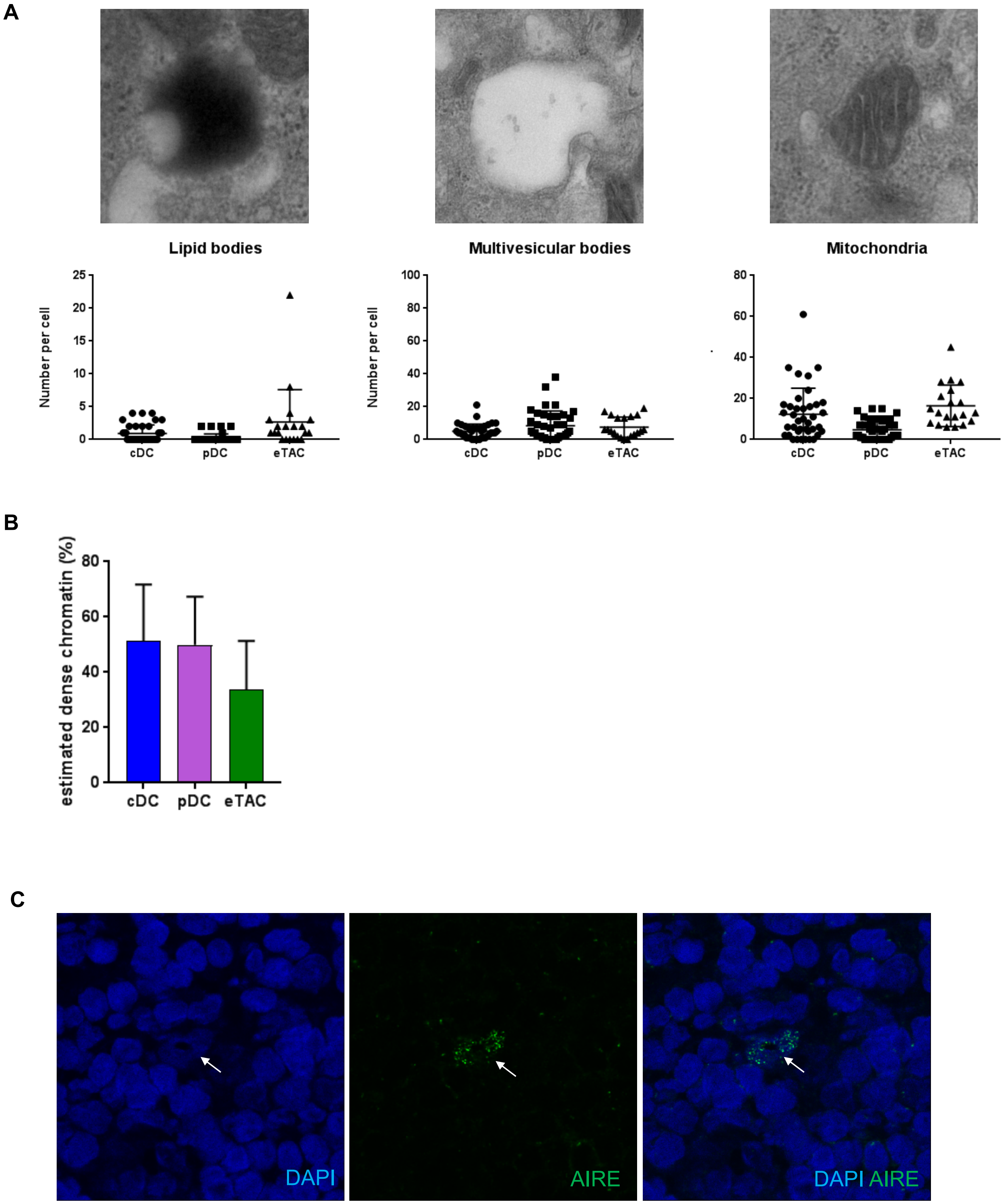

Supplementary Figure 4

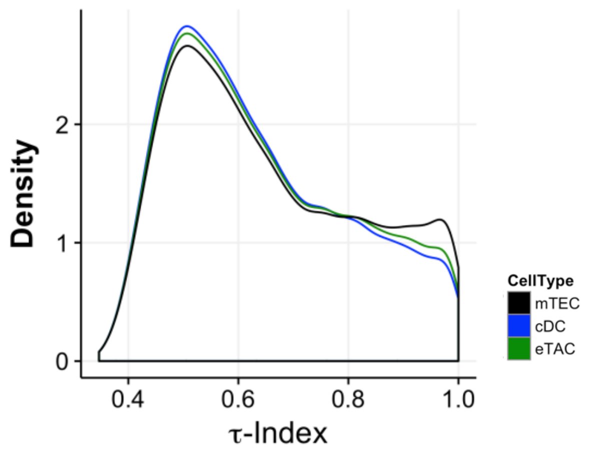

Supplementary Figure 5

**A**

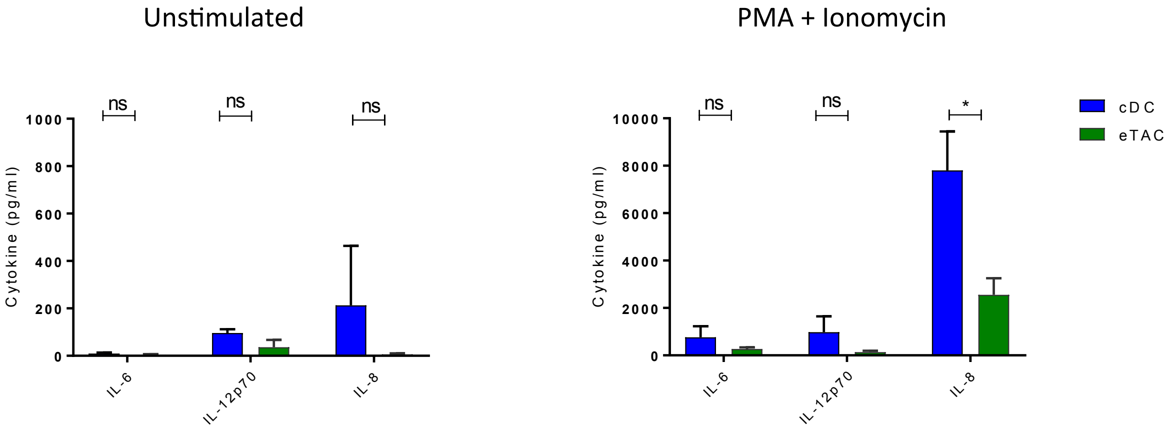


**B**

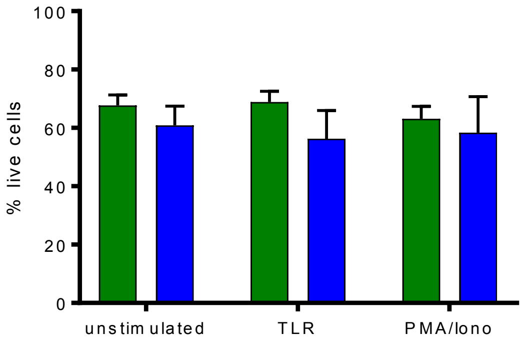


**C**

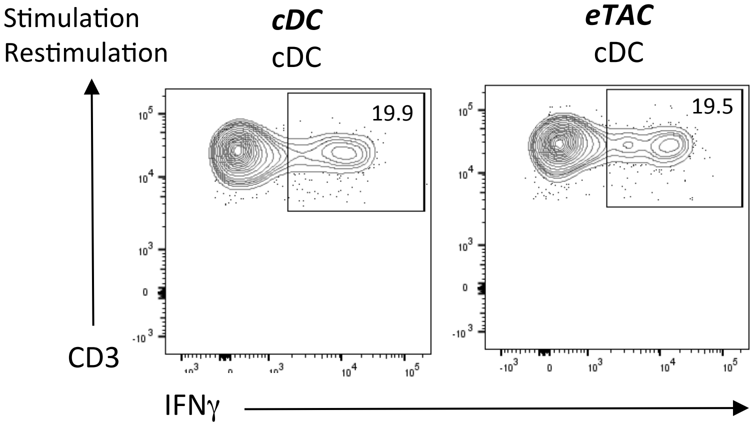

# Supplementary Figure 6

**A** Blue vs. Yellow

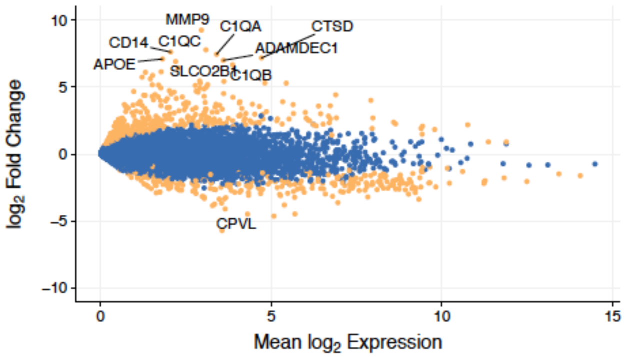


**B** Blue vs. Brown

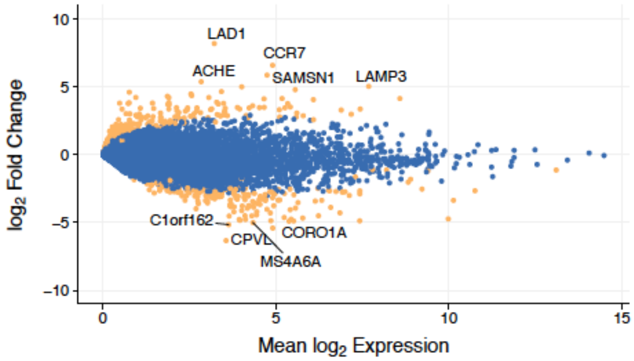


**C** Blue vs. Turquoise

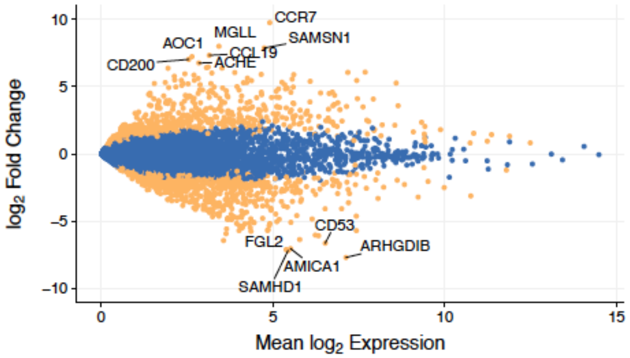

Supplement: Supplementary Figure 1 — Single cell RNA sequencing of tonsil cells reveals AIRE expressing cells. Related to Figure 1. (A) Human thymus section stained by immunofluorescence for DAPI (blue) and AIRE (red), scale bar indicating 10 μM is shown (B) Gating strategy of putative eTACs from tonsil cells depleted of T (CD3) and B (CD19) cells for sorting as single cells for RNA seq analysis (C) Log10 total read counts of all cells according to donor (A; green B; purple) (D) t-SNE plot of single cells from Figure 1E colored according to donors as in b, above (E) Heatmap of the top 75 genes with the strongest influence on separating cluster 1 (blue) and cluster 2 (orange) as shown in Figure 1E and clustered by hierarchical clustering (F) MA plot differential expression testing between clusters identifies CCR7 and CD11c as principal markers of these different DC populations. Plotted are the mean log2 gene expression values (x-axis) against the log2 fold change between clusters. Points below the horizontal are up-regulated in cluster 1 (blue), and those above the horizontal are up-regulated in cluster 2 (orange). Points are colored based on a 1% FDR (red: null hypothesis rejected, gray: failed to reject null hypothesis). [file Data_Sheet_1.PDF]
